# Effects of Sling-Based Thoracic Active Exercise on Pain and Function and Quality of Life in Female Patients with Neck Pain: A Randomized Controlled Trial

**DOI:** 10.3390/healthcare9111514

**Published:** 2021-11-05

**Authors:** Gun-Woo Park, Jungae An, Sang-Woo Kim, Byoung-Hee Lee

**Affiliations:** 1 Graduate School of Physical Therapy, Sahmyook University, Seoul 01795, Korea; ptgunwoo@naver.com (G.-W.P.); jungaean@hotmail.com (J.A.); 2 Virtual Rehabilitation Lab, Sahmyook University, Seoul 01795, Korea; adonis2023@naver.com; 3 Department of Physical Therapy, Sahmyook University, Seoul 01795, Korea

**Keywords:** neck pain, female, thoracic exercise therapy, recovery of function, quality of life

## Abstract

This study aimed to investigate the effects of sling-based thoracic active exercise on pain, function, and quality of life in female patients with neck pain. A total of 27 female patients with neck pain were divided into the sling-based thoracic active exercise group (*n* = 14) and the control group (*n* = 13). The study group performed a sling-based thoracic active exercise with cervical manual therapy for 50 min a day, twice a week for 4 weeks, whereas the control group performed a placebo exercise with cervical manual therapy in the same manner as the study group. Evaluation of the degree of pain before and after treatment was based on the pressure pain threshold and numeric pain rating scale scores. The craniovertebral angle and neck disability index (NDI) were used to evaluate neck function, and quality of life was measured using the Short Form-36. Afterwards, the patients’ pressure pain thresholds were significantly increased, and the numeric pain rating scale score was significantly decreased in both groups (*p* < 0.05). In terms of function, the craniovertebral angle was significantly increased in both groups (*p* < 0.05), and neck dysfunction significantly decreased (*p* < 0.05). The quality of life significantly increased in both groups (*p* < 0.05). The pressure pain threshold, craniovertebral angle, neck dysfunction index, and quality of life scores (*p* < 0.05) were significantly different between groups, except the numeric pain scale score. Our results showed that sling-based thoracic active exercise is effective in reducing pain and improving function and quality of life in female patients with neck pain, thus emphasizing the need for thoracic treatment for such patients.

## 1. Introduction

In the modern world, the use of digital devices and the average daily use time are rapidly increasing, with 81.1% of the Korean adult population using smartphones [1]. Owing to prolonged unstable posture, muscle stiffness, and lack of exercise, 8 out of 10 individuals are at risk for musculoskeletal disorders at least once in their lifetime [2]. Neck pain, a typical musculoskeletal symptom, occurs more than once, and the rate of neck pain in one year in the adult population (aged 15–74 years) is 16.7–75.1%, with a lifetime prevalence of 14.2–71%, and the average rate is 48.5% [3]. In Korea, the number of patients who visited the hospital because of neck pain in 2010–2019 increased by 41% [4], and 15–22% of patients have experienced neck pain for more than 5 years after the onset. Symptoms persist [5], and more than 33% progress to chronic pain [6], which changes not only the overall psycho-emotional state but also the quality of life [7]. Studies on quality of life in relation to chronic diseases and health in Korea also found that neck pain greatly influences the quality of life of patients with musculoskeletal disorders [8].

Compared to men, women have a smaller physique and more adipose tissue; they also have decreased physical strength owing to less muscle development and weak muscle fibers [9], and with aging, their basal metabolic rate, among other physical changes, begins to drop sharply. A sudden decline in physical strength causes loss of muscle mass because of the lack of exercise [10]. In women, the incidence of musculoskeletal symptoms in the upper limbs such as the wrists, shoulders, and fingers is higher than that in men [11], and 60% of all patients with neck pain in Korea are women [4].

Since the physiological cause of chronic neck pain has not been identified, the treatment of neck pain is focused on reducing symptoms such as pain in cases other than specific pain patterns [12,13]. In general, conservative treatments such as hyperthermia, electric stimulation, traction, and laser treatment as physical therapy [14] or interventions applied with manual therapy and exercise therapy [15] are used to reduce pain in patients with epidemiological neck pain.

From an epidemiologic point of view, the association between the cervical and thoracic vertebrae is a fundamental cause of cervical vertebral disorders owing to abnormal movement of the thoracic vertebrae [16]. In the examination of patients with neck pain, the range of motion of the neck and the mobility of the cervical and thoracic segments are limited, and in patients with chronic neck pain, muscle strength and movement control of the cervical, scapular, and thoracic spine appear [17]. Thoracic mobility is important in patients with cervical dysfunction, and manual therapy focused on the thoracic spine improves the range of motion of the cervical spine in patients with forward head posture [18]. The need for thoracic spine treatment is emphasized for neck pain, as studies on the application of manual therapy and joint mobilization to the site are being conducted continuously [18,19].

As an intervention for neck pain, a combination of manual therapy and therapeutic exercise is effective [15]. Active intervention is more effective than passive intervention [20], and active participation has a positive physiological effect [21]. Rather than a passive approach, exercising with a sling is an active exercise technique where the subject actively exercises, and it can also be effectively performed with a closed-chain exercise, which is a suitable method for functional movement or joint stabilization [22]. Closed-chain exercises increase the compression force of the joint through weight-bearing and activate the joint receptor to increase the position recognition of the joint and its effect on related muscles. From a biomechanical point of view, the shear force applied to the joint was compared with open-chain exercises, and a reduction effect was observed [23]. As a result, closed-chain exercises induce cooperative contraction of the agonist and antagonist muscles, thereby increasing joint mobility and stability [24]. In addition, the closed-chain exercise applied to the upper body was suggested as an effective program that strengthens the upper body and provides dynamic stability to the joints [25,26].

Although the sling exercise provides various types of closed-chain exercises for patients to strengthen the musculoskeletal system, and positive benefits have been verified for pain, disability, and quality of life for patients with neck pain, there is insufficient evidence to suggest that active thoracic exercise with a sling is an effective intervention for women with neck pain [27]. Therefore, the aim of this study was to investigate the effects of sling-based thoracic active exercise on pain and function and quality of life in female patients with neck pain.

## 2. Materials and Methods

### 2.1. Participants

This study recruited 33 women who complained of neck pain in the Seoul S Clinic. They fully understood the content of the study, actively expressed their intention to participate, and agreed to participate in the study.

The selection criteria for this study were women aged 30–50 years, pain in one or both sides of the neck, a neck disability index (NDI) score of 24 points or less, and the absence of shoulder and back pain so they could assume the postures necessary for thoracic active exercise with a sling.

Exclusion criteria for the study participants were as follows: neurological symptoms related to the neck; previous surgery on the neck or spine; currently undergoing surgical injection treatment to reduce pain or having sensory abnormalities from neurological abnormalities; muscle paralysis, chest pain, or dizziness; a blood pressure of 160/110 mmHg or higher from high blood pressure, heart disease, or infection; or pregnancy.

The present study was approved by the Institutional Review Board of Sahmyook University (Seoul, Korea, 2-1040781-A-N-012020077HR) and registered (KCT0005487) in the Clinical Research Information Service in the Republic of Korea. The objectives and procedures in the study were fully understood by the participants, who then provided informed consent. Therefore, this study was in accordance with the ethical principles of the Declaration of Helsinki.

### 2.2. Experimental Procedures

**Figure 1:**
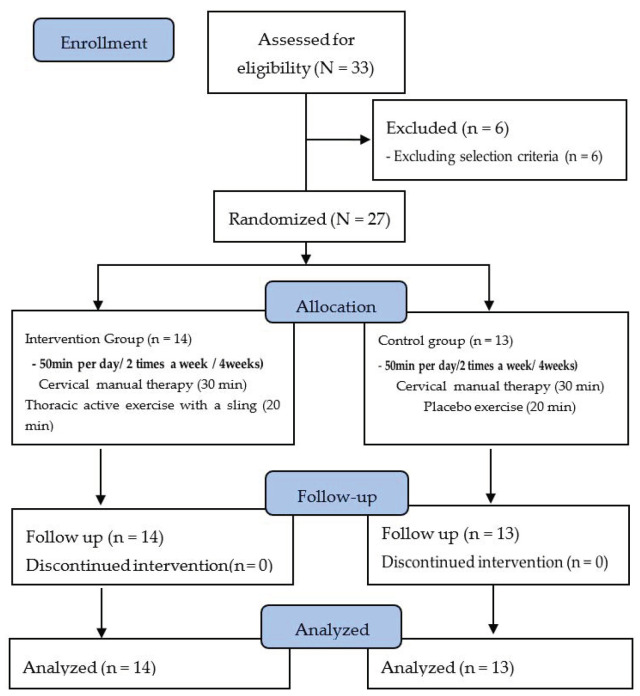
Flow diagram of total experimental procedure.

Approval to access the patients’ medical records was obtained in advance to confirm the patient data related to diagnoses such as clinical characteristics, date of onset, cause of onset, and history, as well as their general characteristics, such as age, weight, height, and BMI. At the time of group selection, bias was minimized by randomly assigning the participants to therapists. Of 33 recruited participants, we excluded 1 because of an NDI score of 24 points or less and 5 because of age. Finally, 27 participants participated in the study, 14 in the sling-based thoracic active exercise (STAE) group and 13 in the control group. The STAE group performed cervical manual therapy for 30 min twice a week for 4 weeks and the thoracic active exercise with a sling for 20 min, while the control group performed cervical manual therapy twice a week for 4 weeks, for 30 min, and a placebo exercise for 20 min. The pre-post evaluation items were pressure pain threshold, numeric pain rating scale score, cranial spine angle, NDI score, and quality of life score. The pre-post evaluations were conducted independently at the same test site by a female physical therapist in consideration of the gender of the study participants. The physical therapist had undergone manual therapy and sling-related training for more than 4 years, practiced manual therapy to be applied to patients in advance, and discussed problems that may arise when applying thoracic active exercises with slings. With sufficient knowledge, treatment was always performed by the same therapist (Figure 1).

#### 2.2.1. Thoracic Active Exercise with a Sling

**Figure 2:**
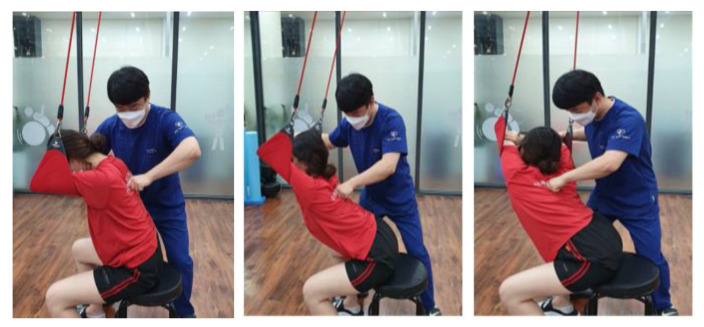
Thoracic active exercise with a sling.

Thoracic active exercise with a sling was applied to women who complained of neck pain as a method of promoting movement of the thoracic joint and inducing active movement of the peripheral muscles through active joint movement in the thoracic region. The STAE program presented in this study was redesigned based on the thoracic mobility exercise presented in the Neurac1 Seminar Workbook [28], and the procedure was as follows (Figure 2).

To perform the thoracic active exercise with a sling, the subject sat under a prepared sling with stable lower limbs and a posture that concentrated the movement on the thoracic spine by adjusting the height of the support band. The subject sat on a chair right under the sling, the feet were placed at the width of the pelvis, with the knees and hips maintained at a 90° angle.

The subject crossed the forearms to naturally place them on the support band, adjusted the length of the string so that the support band could be positioned at the height of the thoracic vertebrae, and lowered the head comfortably to place the forehead in the middle of the crossed forearms. For the first motion, the flexion and extension motion, the support band of the thoracic spine was gently pressed, the elbow was extended forward, and the thoracic spine was extended. In the extended state, the upper limbs supported by the support band were pulled, and the thoracic spine was flexed to enable active movement. The subject actively repeated the movement, and the therapist, positioned behind the subject, applied joint mobilization to the thoracic region.

In the second motion, the left and right flexion motion, the subject pressed the support belt lightly and moved the body from side to side while leaning on the support belt to perform lateral flexion of the thoracic vertebrae; the subject actively repeated the movement, and the therapist moved the joint on the thoracic vertebrae while positioned behind the subject.

Each exercise was performed for 20 min in three sets of 15 repetitions. Over the 4 weeks of the experiment, the amount of joint mobilization applied by the therapist each week was gradually reduced so that the active movement of the subject increased (Table 1).

#### 2.2.2. Manual Therapy

Manual therapy of the neck involved soft tissue relaxation, joint mobilization, and passive stretching based on the manual suggested by Ylinen et al. [29]. Passive stretching was performed on the periphery of the cervical spine (upper trapezius, pectoralis major, pectoralis minor, etc.) for 5 min, followed by soft tissue relaxation on the upper trapezius, suboccipital muscle, cervical flexor, rhomboid muscle, central trapezius, scalenes, sternocleidomastoid muscle, pectoralis major, and pectoralis minor. This lasted for a total of 15 min, followed by 5 min of joint mobilization on the cervical vertebrae, and then passive stretching for 5 min of the cervical vertebrae (scalenes, nuchal ligament, etc.). This therapy lasted for a total of 30 min per day, twice a week, for 4 weeks (Table 2).

#### 2.2.3. Placebo Exercise

The placebo exercise was performed as a placebo motion of the thoracic vertebrae applied during the thoracic active exercise with a sling. The subject sat upright on a chair and repeatedly performed a simple motion, which was the range of motion of the thoracic spine flexion and extension and lateral flexion. The exercise was performed for 20 min twice a week for 4 weeks, with each movement repeated for three sets of 15 repetitions.

### 2.3. Outcome Measurements

In this study, pain was measured using a pressure pain threshold and numeric pain rating scale. The pressure pain threshold was measured in N units using a pressure pain gauge (Wagner Force Ten-FDX; Wagner Instrument, CT, USA, 2010). The measurement sites were the splenius capitis, upper trapezius, and middle trapezius muscles; when the pressure pain threshold was measured, the participants were comfortably placed in a prone position to maintain a neutral posture. The cervical parietal muscle was measured 1 cm away from the fourth cervical vertebra, and the gastric triceps muscle was measured perpendicular to the skin surface by marking the midpoint between the spinous process and the acromion process of the seventh cervical spine. The medial dorsal triceps were measured after marking the midpoint of the shoulder bone pole and the midpoint of the spine at this height. During measurement, an electronic pressure gauge was placed vertically on the surface of the skin, and a pressure of 1 kg/s was gradually applied to the patient; when they felt pain or discomfort, they said “top,” three times, which was then divided by the average value. The intra-meter (r = 0.85) and inter-meter reliabilities (r = 0.85) of the pressure statistics were very high [30].

A numeric pain rating scale was used, with 0 for no pain and 10 for unbearable pain. This method expressed the degree of pain in a simple and reproducible manner where the patient conveyed the level of pain by displaying the average value felt for 24 h. It had a high sensitivity and high reliability (ICC = 0.90) [31].

In this study, function was measured using the craniovertebral angle and the NDI. The craniovertebral angle is the angle of the line connecting the ear migration and the seventh neck bone and the horizontal line passing through the seventh neck bone and the horizontal line passing through the seventh cervical vertebra [32], and it was measured using a body shape measurement and analysis system (Gait & Posture Assessment System 500; Alfoots, KOR, Seoul, Korea, 2017). During the measurement, the subject was photographed in a natural standing position, and then the value was measured with the analysis program of the device. Before the measurement, while the subject was in the sitting position, the examiner guided the flexion and extension of the subject’s cervical spine while holding the subject’s forehead with two fingers at the C6 and C7 spinous processes. If spinous processes were not supported by the examiner’s finger during the extension of the cervical spine, the affected area was judged as C6, and the stimulated spinous process was judged as C7. Subsequently, the inspector attached a labeling sticker to the C7 spinous process. The subject was photographed after taking five steps in place and then assuming a natural standing position. After taking the picture, a body shape measurement analysis program was used to quantify the craniovertebral angle of the subject. This test showed high reliability (ICC = 0.88) [33].

The NDI is a self-assessment tool developed to evaluate the daily life performance of patients with neck pain. It consists of 10 questions: pain intensity, lifting, concentration, reading, headache, self-care, driving, work, sleep, and leisure activities [34]. The score for each question was 0–5 points, and the total score was recorded by adding the scores for all questions. In this test, the reliability of ICC = 0.93, and Cronbach’s alpha value of internal consistency was 0.82, showing high reliability and validity [35].

In this study, the quality of life was measured using the general health-related quality of life measurement tool Short Form-36 (SF-36). SF-36 has eight scales: physical functioning, role-physical, bodily pain, general health, vitality, social functioning, and emotional, which consists of role-emotional and mental health. Data values of the eight scales were converted to obtain a value between 0 and 100, and the higher the score, the better the health status. The first four scales were grouped in a physical component summary (PCS), and the latter four scales were grouped in a mental component summary (MCS). The two values of physical and mental factors were collectively called global health (GH). The higher the score, the higher the quality of life in the area. The range of reliability for retesting of the Korean SF-36 was r = 0.710–0.895, and the range of Cronbach’s alpha for internal consistency was 0.930–0.938 [36].

### 2.4. Statistical Analysis

SPSS statistical software (IBM, Chicago, IL, USA), version 23.0, was used for all statistical analyses. The Shapiro–Wilk test was used to analyze the normal distribution of the variables. An independent samples *t*-test was performed to identify differences between the groups. The paired *t*-test was used to compare the results before and after the intervention. For all tests, the level of statistical significance was set at *p* < 0.05.

## 3. Results

The demographic characteristics are shown in Table 3. No significant differences were observed in the baseline values between the STAE group and the control group for all parameters.

### 3.1. Pain

The results of the comparison of the pre-post test measures of the pressure pain threshold of the splenius capitis and upper and middle trapezius muscles between the two groups are shown in Table 4. The pressure pain thresholds on both sides of the splenius capitis muscle, both sides of the upper trapezius muscle, and both sides of the middle trapezius muscle were significantly improved after the intervention within the STAE group and the control group (*p* < 0.05), and the STAE group showed significant improvement compared to that in the control group (*p* < 0.05).

The results of the comparison of pre-post test measures of the numeric pain rating scale scores between the two groups are shown in Table 5. The pain rating was significantly improved after the intervention in the STAE and control groups (*p* < 0.05), However, no significant difference was observed in the value of the pre-post difference between groups.

### 3.2. Function

The results of the comparison of the pre-post test measures of the craniovertebral angle in neck function between the two groups are shown in Table 6. The craniovertebral angle was significantly improved after the intervention in the STAE and control groups (*p* < 0.05), and the STAE group showed significant improvement compared to that in the control group (*p* < 0.05).

The NDI score was significantly improved after the intervention within the STAE group and control groups (*p* < 0.05), and the STAE group showed significant improvement compared to that in the control group (*p* < 0.05).

### 3.3. Quality of Life

The results of the comparison of pre-post test measures of PCS, MCS, and GH in the quality of life score between the two groups are shown in Table 7. The PCS, MCS, and GH were significantly improved after the intervention in the STAE and control groups (*p* < 0.05), and the STAE group showed significant improvement compared to that in the control group (*p* < 0.05).

## 4. Discussion

The aim of this study was to investigate the effects of sling-based thoracic active exercise on pain, function, and quality of life in female patients with neck pain. Our results showed that sling-based thoracic active exercise is effective in reducing pain and improving function and quality of life in female patients with neck pain.

In the case of neck pain with limited mobility, neck pain appears in the center or at one side of the neck and is associated with pain in the shoulder and upper limbs [17]. In addition, decreased mobility of the upper thoracic spine affects the load and mobility of the cervical spine [37]. As a result, decreased mobility of the thoracic spine causes increased pain around the neck [38]. In this study, the pressure pain threshold (PPT) was significantly increased after intervention in both group (*p* < 0.05), and a significant difference was also observed in the comparison of the differences between groups according to treatment methods (*p* < 0.05). The numeric pain rating scale score was significantly reduced from 7.64 to 3.43 points in the thoracic active exercise with a sling group after treatment (*p* < 0.05) and significantly decreased in the control group from 7.38 to 4.08 points after treatment (*p* < 0.05). Celenay et al. [39] conducted a study on the effectiveness of the combined treatment of cervical and thoracic shoulder exercises and connective tissue massage in 62 patients with mechanical neck pain. There was a significant difference within the group (*p* < 0.05) and between groups (*p* < 0.05) with the increase in the upper trapezius muscle from 75.8 to 98.7 N and the left upper trapezius muscle from 69.09 to 96.04 N. Pain decreased when the pressure on the joint decreased and when the space between the two joint surfaces increased. In a study by Masaracchio et al. [40], in which a combination of thoracic orthodontic treatments was applied to 64 patients with mechanical neck pain, the numerical pain rating scale scores significantly decreased from 5.1 to 2.2 points in the experimental group and from 4.9 to 3.5 points in the control group (*p* < 0.05). These results were consistent with those of previous studies, but there was a greater decrease in thoracic active exercise with slings in the comparison of differences between groups according to treatment methods. The difference was not statistically significant, so the results were partially inconsistent with previous studies. Compared with previous studies, this study was consistent with the decrease in the numerical pain rating scale scores within the groups, but there was no statistically significant difference because of the small number of participants, and it was concluded that there is a need for supplementation in subsequent studies.

The ideal alignment occurs when the head posture does not tilt upward or downward, does not tilt sideways, and does not rotate, and the chin is not protracted [41]. Falla et al. [42] used the craniovertebral angle to measure head posture as a method of measuring abnormal neck alignment. It has been reported that the craniovertebral angle is accompanied by pain when it decreases by less than 50° [43]. It has also been suggested that those with a relatively small craniovertebral angle have a higher rate of neck pain than those with a relatively large craniovertebral angle [32]. In addition, in patients with chronic neck pain, functional movements of the upper limb resulted in a change in the muscle activation pattern, and the muscle tone of the sternocleidomastoid, scalenus anterior, and upper trapezius muscles increased. When the craniovertebral angle is increased because of abnormal alignment of the cervical vertebra, the length changes, and hypertension of the upper trapezius muscle, levator scapula, sternoclavicular muscle, and scalene muscle occurs, and weakness of the lower trapezius, latissimus dorsi, and shoulder muscles is experienced [44,45]. As a result of measuring the change in neck alignment in this study, thoracic active exercise with slings increased the angle significantly from 59.84° to 63.16° after treatment (*p* < 0.05). There were also significant differences in the comparison of differences between groups according to the treatment methods (*p* < 0.05). In a previous study, Cho et al. [46] applied upper thoracic mobilization and cervical mobilization to the experimental group to compare the effects of upper thoracic mobilization in 32 patients with anterior head posture. Upper cervical mobilization and stability exercises were performed in the control group. In this study, the craniovertebral angle increased from 48.8° to 54.1° in the experimental group and from 49.8° to 51.1° in the exercise group, showing significant differences within the experimental group (*p* < 0.05). There were no significant differences between groups.

In patients with chronic neck pain, decreased mobility of the spine and chest cage appears, and various neck dysfunctions occurs for this reason [47]. It has been reported that an increase in the craniovertebral angle causes hyperflexion of the lower cervical vertebrae and hyperextension of the upper cervical vertebrae. As a result, musculoskeletal stress in the neck region increases, resulting in clinical symptoms [48]. It is thought that the enhancement of thoracic vertebrae movement through active thoracic vertebrae exercise with a sling affects the alignment of the neck to improve movement. The improved movement affects the muscles and joints around the neck and back, and the improved thoracic joint movement is thought to have affected the movement and alignment around the neck. As a result, the thoracic active exercise combined with a sling including an active element is considered a more effective intervention in improving the craniovertebral angle in female patients with neck pain than passive mobilization.

Based on our results, thoracic active exercise with a sling decreased the NDI score significantly from 17.50 to 9.57 points after treatment (*p* < 0.05). There were also significant differences between groups based on the treatment methods (*p* < 0.05). In a previous study, Arsh et al. [49] reported that in a study in which manual therapy was applied to the upper thoracic spine in 37 nonspecific neck pain patients, the NDI score decreased from 20.4 to 7.50 points in the experimental group and from 23.8 to 12.4 points in the control group. There was a significant difference between groups (*p* < 0.05). The results of this study were consistent with those of previous studies.

If abnormal neck alignment continues, the amount of cervical spine flexion increases. At the same time, the activity of the cervical extensor muscles increases to maintain balance, leading to an increase in the activity of the upper trapezius muscles [50]. This causes muscle contraction in the cervical extensors, upper trapezius, and levator scapula, which are the affected muscles, and relaxation of the cervical flexors [41]. Among these muscles, muscle pain occurs in the area of muscle contraction, causing musculoskeletal pain around the neck and shoulders [51]. The decrease in function can be seen as a result of neck pain and neck alignment change and decreased movement; mechanically, the back and neck are correlated. It appears that the thoracic active exercise with sling performed in this study resulted in a change in muscle activation pattern and functional movement of the thoracic and cervical vertebrae, resulting in a decrease in the NDI score. Therefore, the improved thoracic spine movements might have influenced the recovery of normal movements of the neck and peripheral muscles, thereby enhancing function.

Based on the SF-36 test, the physical factor increased the quality of life points significantly from 40.22 to 70.37% after treatment with thoracic active exercise with sling (*p* < 0.05). The mental factor increased significantly from 43.12 to 67.46% after treatment (*p* < 0.05). Overall health, which was the sum of physical and mental factors, increased significantly from 41.67 to 68.92% after treatment (*p* < 0.05). There were significant differences between groups according to the treatment methods (*p* < 0.05). Celenay et al. [39] conducted a comparative study of 102 patients with nonspecific mechanical neck pain in which stabilization exercise and manual therapy were combined or only stabilization exercise was performed. In this experimental group, the physical factor increased from 33.9 to 40.9 points, and the mental factor increased from 42.6 to 48.9 points, showing a significant difference within the group (*p* < 0.05). In the control group, there was a significant difference (*p* < 0.05), from 37.0 to 41.0 points for the physical factor and from 41.9 to 44.8 points for the mental factor. Individuals with pain may experience mental confusion and have poor health-related quality of life [52]. A low quality of life score can lead to a major depressive disorder [53], and depression, which can result from poor quality of life, is the most common mental disorder worldwide and one of the 10 major causes of disability [54]. Decreased quality of life leads to a more sensitive response to pain by increasing the effect of pain on social and occupational functioning [55]. Individual achievement and emotional satisfaction in performing functional activities in daily life or processes are important factors that determine the quality of life. Neck pain was alleviated through thoracic active exercise with a sling; the quantity and quality of movement was improved through the improvement of neck function, and the increased activity increased the subjective life satisfaction of the participants. Physical and mental factors were thought to have improved. The combination of passive joint mobilization, called thoracic active exercise with a sling, and the lack of active participation of the patient occur when only passive treatments such as manipulation and joint mobilization are performed. This is a complementary point. Thus, the combined approach is an effective intervention for improving the quality of life of patients with neck pain.

The limitations of this study were that in the selection of participants, patients who currently visited the hospital for pain and received treatment were targeted; they were limited to specific sexes, and the number of participants was small. This made it difficult to generalize the results to all patients with neck pain. In addition, regarding the application and effect of the treatment, the effect was confirmed after eight treatments twice a week for 4 weeks; however, long-term treatment was not performed, and no studies were conducted after the treatment was terminated. In future studies, taking into account these limitations, it is recommended that thoracic active exercise with a sling should be applied to large sample size, and interventions mediated through a long-term treatment plan.

## 5. Conclusions

This study aimed to investigate the effect of thoracic active exercise with a sling on pain, function, and quality of life in female patients with neck pain. Significant differences were observed in the pain, function, and quality of life of the pressure pain threshold between the groups. In future studies, a study with a large sample size is required for comprehensive application of thoracic active exercise using a sling as an effective intervention for neck pain.

## Figures and Tables

**Table 1:** Thoracic active exercise with sling.

Week	Weekly Training	Times
1 Week	Flexion-Extension, Lateral Flexion Therapist’s Joint Exercise Guide (Passive Joint Mobilization)	15 times, 3 set
2 Week	Flexion-Extension, Lateral Flexion Therapist’s Joint Exercise Guide (Passive Joint Mobilization 50%)	
3 Week	Flexion-Extension, Lateral Flexion (Passive Joint Mobilization 30%)	
4 Week	Flexion-Extension, Lateral Flexion (Passive Joint Mobilization 15%)	

**Table 2:** Manual therapy process.

Technique	Manual Therapy	Time
Passive stretching	periphery of the cervical spine (upper trapezius, pectoralis major, pectoralis minor, etc.)	5 min
Soft tissue relaxation	upper trapezius, suboccipital muscle, cervical flexor, rhomboid muscle, central trapezius, scalenes, sternocleidomastoid muscle, pectoralis major, and pectoralis minor	15 min
Joint mobilization	cervical vertebrae	5 min
Passive stretching	cervical vertebrae (scalenes, nuchal ligament, etc.)	5 min

**Table 3:** Demographic data of groups (N = 27).

	STAET Group (*n* = 14)	Control Group (*n* = 13)	*t*(p)
Age(year)	43.21(7.96)	46.77(9.10)	−1.082(0.290)
Height(cm)	158.86(3.84)	159.69(3.54)	−0.586(0.563)
Weight(kg)	57.00(4.00)	54.15(3.00)	2.079(0.048)
BMI	22.63(2.07)	21.28(1.80)	1.801(0.084)

Values are means (SD). STAE = sling-based thoracic active exercise; BMI = body mass index.

**Table 4:** Comparison of pressure pain threshold (N = 27).

Parameters		STAET Group (*n* = 14)	Control Group (*n* = 13)	*t*(p)
Splenius capitis (Right)	Pre	19.96(3.95)	20.04(4.93)	−0.047(0.963)
	Post	36.34(5.53)	31.35(5.89)	
	Pre-post	−16.38(5.43)	−11.32(5.32)	−2.447(0.022) *
	*t*(p)	−11.290(0.000) *	−7.665(0.000) *	
Splenius capitis (Left)	Pre	20.13(4.26)	19.18(4.48)	0.561(0.579)
	Post	34.86(4.67)	29.95(5.90)	
	Pre-post	−14.73(4.59)	−10.77(4.26)	−2.319(0.029) *
	*t*(p)	−12.014(0.000) *	−9.117(0.000) *	
Upper trapezius (Right)	Pre	21.59(4.28)	21.97(6.38)	−0.185(0.855)
	Post	37.61(4.51)	30.56(6.61)	
	Pre-post	−16.02(4.63)	−8.59(4.37)	−4.278(0.000) *
	*t*(p)	−12.939(0.000) *	−7.090(0.000) *	
Upper trapezius (Left)	Pre	19.34(5.14)	21.00(3.53)	0.968(0.342)
	Post	36.79(5.46)	29.75(4.47)	
	Pre-post	−17.45(4.39)	−8.75(4.73)	−4.958(0.000) *
	*t*(p)	−14.881(0.000) *	−6.676(0.000) *	
Middle trapezius (Right)	Pre	23.68(6.29)	28.64(7.11)	−1.9220.066)
	Post	43.65(8.05)	33.52(7.25)	
	Pre-post	−19.97(8.63)	−4.88(3.56)	−6.012(0.000) *
	*t*(p)	−8.658(0.000) *	−4.5943(0.000) *	
Middle trapezius (Left)	Pre	23.81(5.55)	28.27(5.26)	−2.137(0.043)
	Post	42.96(6.46)	33.06(5.64)	
	Pre-post	−19.14(6.71)	−4.79(3.65)	−6.82(0.000) *
	*t*(p)	−10.676(0.000) *	−4.736(0.000) *	

Values are means (SD). STAE = sling-based thoracic active exercise, * *p* < 0.05.

**Table 5:** Comparison of numeric pain rating scale (N = 27).

Parameters		STAET Group (*n* = 14)	Control Group (*n* = 13)	*t*(p)
NPRS (Point)	Pre	7.64(0.84^)^	7.38(1.04)	0.710(0.484)
	Post	3.43(0.76)	4.08(0.76)	
	Pre-post	4.21(1.12)	3.31(1.25)	1.986(0.058)
	*t*(p)	14.057(0.000) *	9.536(0.000) *	

Values are means (SD). STAE = sling-based thoracic active exercise; NPRS = numeric pain rating scale, * *p* < 0.05.

**Table 6:** Comparison of craniovertebral angle (N = 27).

Parameters		STAET Group (*n* = 14)	Control Group (*n* = 13)	*t*(p)
CVA (°)	Pre	59.84(3.00)	59.19(2.17)	−0.639(0.529)
	Post	63.16(2.42)	60.03(2.06)	
	Pre-post	−3.31(1.08)	−0.84(0.77)	−8.088(0.000) *
	*t*(p)	−11.492(0.000) *	−3.919(0.002)	
NDI (Point)	Pre	17.50(2.93)	16.62(3.15)	0.6754(0.458)
	Post	9.57(1.65)	12.15(2.15)	
	Pre-post	7.93(2.30)	4.46(1.81)	4.328(0.000) *
	*t*(p)	12.883(0.000) *	8.897(0.000) *	

Values are means (SD). STAE = sling-based thoracic active exercise; CVA = craniovertebral angle; NDI = neck disability index, * *p* < 0.05.

**Table 7:** Comparison of quality of life (N = 27).

Parameters		STAET Group (*n* = 14)	Control Group (*n* =13)	*t*(*p*)
PCS (Point)	Pre	40.22(12.13)	48.65(11.61)	−1.845(0.077)
	Post	70.37(11.45)	57.79(12.57)	
	Pre-post	−30.15(12.46)	−9.13(5.53)	−5.732(0.000) *
	*t*(*p*)	−9.055(0.000) *	−5.957(0.000) *	
MCS (Point)	Pre	43.12(10.99)	50.32(11.36)	−1.674(0.107)
	Post	67.46(9.73)	56.44(14.23)	
	Pre-post	−24.34(8.43)	−6.12(6.42)	−6.284(0.000) *
	*t*(*p*)	−10.810(0.000) *	−3.440(0.005)	
GH (Point)	Pre	41.67(10.37)	49.49(10.41)	−1.953(0.062)
	Post	68.92(7.01)	57.11(12.73)	
	Pre-post	−27.25(7.75)	−7.63(4.93)	−7.779(0.000) *
	*t*(*p*)	−13.156(0.000) *	−5.582(0.000) *	

Values are means (SD). STAE = sling-based thoracic active exercise; PCS = physical component summary; MCS = mental component summary; GH = global health, * *p* < 0.05.

## Data Availability

Not applicable.
